# Atypical Presentation of Atypical Teratoid Rhabdoid Tumor in a Child

**DOI:** 10.1155/2013/815923

**Published:** 2013-05-27

**Authors:** Y. T. Udaka, K. Shayan, N. A. Chuang, J. R. Crawford

**Affiliations:** ^1^The Department of Pediatrics, University of California, San Diego and Rady Children's Hospital, 3020 Children's Way, San Diego, CA 92123, USA; ^2^The Department of Pathology, University of California, San Diego and Rady Children's Hospital, 3020 Children's Way, San Diego, CA 92123, USA; ^3^The Department of Radiology, University of California, San Diego and Rady Children's Hospital, 3020 Children's Way, San Diego, CA 92123, USA; ^4^The Department of Neurosciences, University of California, San Diego and Rady Children's Hospital, 3020 Children's Way, San Diego, CA 92123, USA; ^5^University of California, San Diego, Division of Child Neurology, Rady Children's Hospital, 8010 Frost Street Suite 400, San Diego, CA 92123, USA

## Abstract

Atypical Teratoid Rhabdoid Tumor (ATRT) is a rare malignant intracranial neoplasm more commonly diagnosed in young children. The authors report the case of an 11-year-old boy with a long standing history of slowly progressive weight loss, fatigue, and weakness over 1.5 years whose magnetic resonance imaging revealed a large heterogeneous enhancing dorsally exophytic lower brainstem mass. Examination revealed extreme cachexia, gaze-evoked nystagmus, dysphagia, dysarthria, bilateral dysmetria, and global weakness without ambulation. The protracted history and neuroimaging features were most suggestive of a low grade glioma. However, pathology revealed a hypercellular tumor with large hyperchromatic nucleoli and loss of INI-1 staining on immunohistochemistry consistent with a diagnosis of an ATRT. The child died shortly after surgery due to complications from his brainstem infiltrative disease. This case illustrates the diverse presentation of ATRT in childhood that can clinically and radiographically mimic that of low grade glioma.

## 1. Introduction

Primary childhood brain and central nervous system (CNS) brain tumors occur at an incidence of 5.1 per 100,000 in the United States with a slightly higher rate in males [1]. It is the second most common malignancy and the most common form of solid tumors in children. Atypical Teratoid Rhabdoid Tumor (ATRT) is a rare malignant intracranial neoplasm occurring mainly in young children comprising only 1-2% of all pediatric brain tumors but approximately 10–20% of CNS tumors in patients less than 3 years of age [2, 3]. Primary CNS ATRT was recognized as a separate entity and added to the World Health Organization (WHO) tumor classification of tumors in 1993 as a grade IV embryonal tumor [4, 5]. ATRT is a highly aggressive tumor that was previously misclassified as a medulloblastoma or primary neuroectodermal tumor (PNET) until being described as a discrete clinical entity in the 1980s. ATRT can occur in both supratentorial and infratentorial regions and often is associated with poor survival depending on the location and degree of resection. Immunohistochemistry plays a role in confirming the diagnosis with loss of INI-1 staining in the neoplastic cells [6]. We report an 11-year-old child who had an atypical clinical presentation and radiographic features for an ATRT. It is important for clinicians to recognize the diverse presentation of ATRT that can mimic that of low grade gliomas.

## 2. Case Report

**Figure 1:**
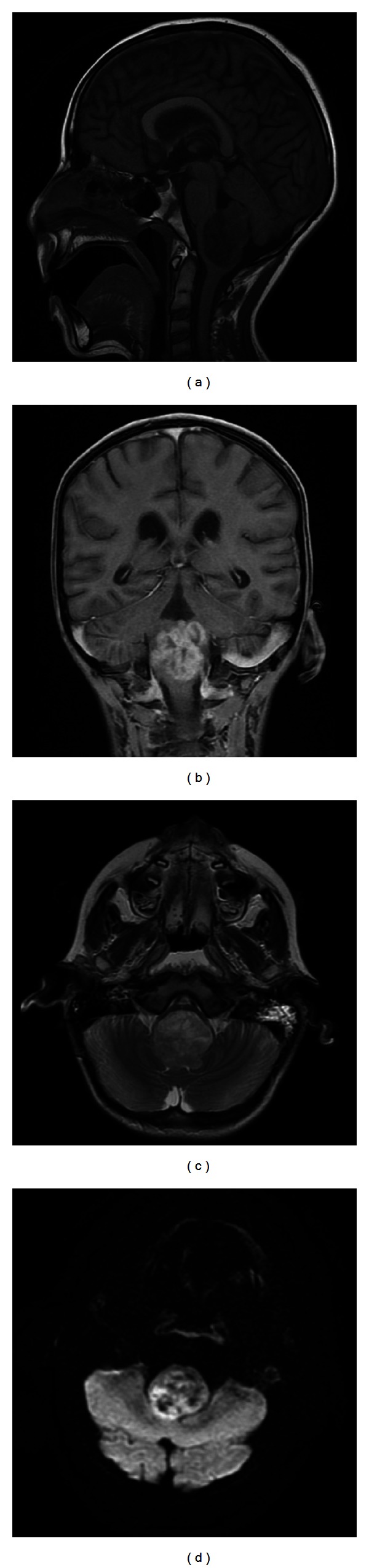
MRI features of Atypical Teratoid Rhabdoid Tumor. Sagittal T1 MRI demonstrates a large heterogeneous dorsally exophytic lower brainstem mass (a) which shows robust enhancement on postcontrast coronal T1 sequence (b). The tumor causes extensive compression of the ventral medulla on transaxial T2 sequence (c) and areas of reduced diffusivity (high signal intensity) on transaxial diffusion-weighted sequence (d).

**Figure 2:**
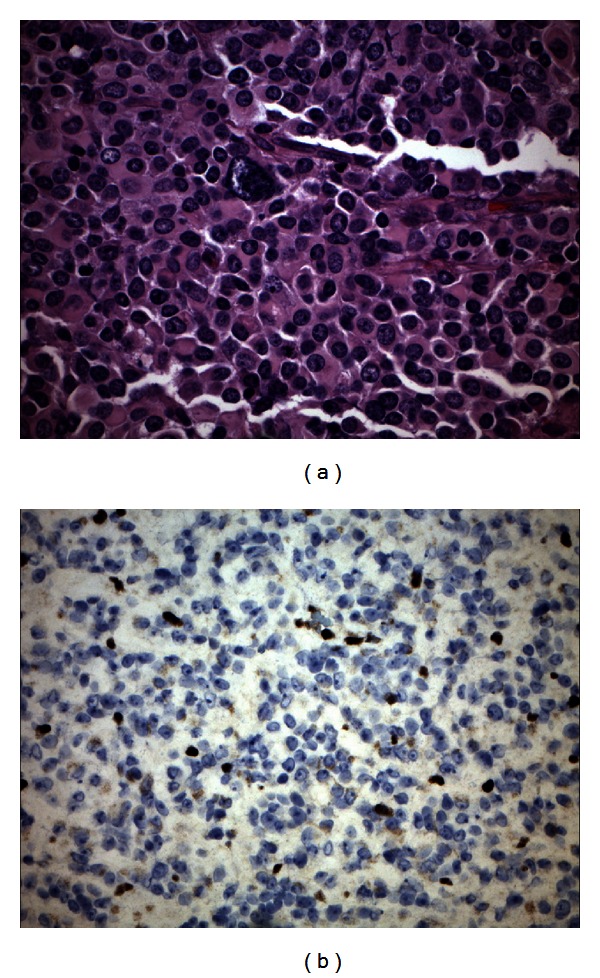
Histologic features of Atypical Teratoid Rhabdoid Tumor. (a) Histologic examination of the tumor reveals diffuse eosinophilic cytoplasmic globules, vesicular chromatin, and scattered large pleomorphic nucleoli (hematoxylin and eosin 40x). (b) Loss of INI-1 expression of the neoplastic cells by immunohistochemistry staining confirms the diagnosis of ATRT (40x).

An 11-year-old previously healthy male presented to our hospital following a one-and-a-half year history of worsening vomiting, extreme cachexia, dysphagia, drooling, and a rapid decline with progressive weakness to the point of inability to ambulate over a course of 2-3 months. His neurological examination at presentation revealed bilateral lateral and down gaze nystagmus, diminished strength most noticeable in finger and wrist extensors of the right upper extremity, and mildly decreased rapid alternating movements of right upper and lower extremities. Magnetic resonance imaging (MRI) examination of the brain revealed a large heterogeneous enhancing mass on T2-weighted imaging centered in the medulla with dorsal exophytic extension into the fourth ventricle with internal areas of restricted diffusion but relative lack of surrounding edema (Figure 1). Internal areas of low signal intensity on susceptibility-weighted imaging likely represented hemorrhage and/or calcification. Given the patient's age, chronicity of symptoms, tumor location, and appearance on MRI, a low grade glioma was suspected and a subtotal resection was obtained. Microscopic examination revealed a round blue cell tumor of high cellularity composed of atypical cells with eccentric nuclei, small nucleoli, and abundant amounts of eosinophilic cytoplasm with frequent mitotic figures. Immunohistochemistry studies revealed loss of INI-1 staining in neoplastic cells confirming the diagnosis of ATRT (Figure 2). The patient died two months following surgery due to complications of his brainstem infiltrative disease.

## 3. Discussion

ATRT remains a significant challenge in pediatric neurooncology. Due to its aggressive nature and high rate of leptomeningeal dissemination, children often progress within months to a year of diagnosis [7, 8]. Data from the ATRT tumor registry estimate that approximately 20% of patients have disseminated disease at the time of presentation [7]. Our reported case is unusual in that our patient had progressive symptoms for a year and a half without dissemination, hydrocephalus, or significant intratumoral hemorrhage, suggesting a more indolent course than what is typical for this highly malignant CNS tumor. His age at presentation was atypical given the higher predilection of this tumor type to children less than 3 years of age. However, although rare, ATRTs have also been reported in adult patients [9]. There are no specific imaging features for intracranial ATRTs [10, 11]. They are more often intraaxially positioned but can be either supra- or infratentorial in location and usually show reduced diffusion on MRI. Supratentorial ATRT can exhibit a cystic appearance with a heterogeneously enhancing wall in some cases [12]. Given the high tendency for CNS dissemination a contrast-enhanced MRI of the entire spine is essential for staging because the presence of disseminated leptomeningeal disease is associated with a significantly higher mortality rate [10]. Although not specific to ATRTs and not statistically significant, ATRTs have a higher tendency for being large and often have some component of calcification, hemorrhage, or necrosis on imaging [11]. In our patient the tumor did display some of these nonspecific characteristics of an ATRT. However, it was surprising given, the long standing history of progressive symptoms, that neither leptomeningeal dissemination nor hydrocephalus was present.

ATRTs can mimic supratentorial PNET and medulloblastoma both histologically and radiographically. One of the earliest studies looking at a series of infants with CNS ATRTs showed an association with an abnormality on chromosome 22 [8, 13]. ATRTs are characterized by a loss of the long arm of chromosome 22 which results in a loss of the hSNF5/INI-1 gene [3]. This has become the defining molecular signature of ATRT. Molecular advances in our understanding of ATRT have made it possible to distinguish it from PNET and medulloblastoma by the absence of INI-1 immunohistochemical staining. Although ATRTs are rare tumors especially in the older pediatric population, it is important to recognize the diverse presentation and radiographic features that may mimic those of low grade glial neoplasms.
